# Income-related inequality in obesity and its determinants in Spain: What happens beyond the obesity threshold?

**DOI:** 10.1007/s10754-023-09360-1

**Published:** 2023-08-03

**Authors:** Athina Raftopoulou, Joan Gil Trasfi

**Affiliations:** 1 Department of Economics, University of Patras, 265 04 Rio Patras, University Campus Greece; 2 Department of Economics and BEAT, Universitat de Barcelona, Barcelona, Spain

**Keywords:** Income-related inequalities, Obesity status, Decomposition analysis, Obesity depth and severity, I12, I14, I18

## Abstract

This paper computes and decomposes income-related inequalities in three metrics of obesity, namely, status, depth and severity, for Spain, a European country characterized by a universal health care system with very high and rising obesity prevalence rates. Furthermore, this paper investigates the main determinants of the reduction in obesity inequalities observed over time among the female Spanish population. To compute these inequality indexes, we use cross-sectional and individual-level data gathered from the Spanish National Health Survey. We document income-related inequalities in obesity, that are more pronounced in depth and severity and are to the detriment of poor women in Spain. University education is the most important determinant for all three inequality indexes. We further report that inequalities in obesity tend to decline over time for women, which is explained mainly by a substantial decrease in the degree of inequality in secondary education and a large decrease in the income elasticity of obesity.

## Introduction

The world is threatened by a huge epidemic of obesity with data showing disappointing trends in the coming years. Paralleling the obesity crisis in the US, Europe is confronted by a similar obesity challenge and although researchers and health authorities argue that they have advanced their understanding of this epidemic, obesity rates still remain at extremely alarming levels. According to the OECD (2019), over half the adult Spanish population is overweight leading to an average reduction of around 2.6 years in life expectancy. Overweight accounts for 9.7% of health expenditure and lowers labor market outputs, resulting in a reduction in Spain’s GDP of 2.9% (OECD, 2019). Moreover, large disparities in obesity exist between the poor and better-off in Spain, especially among women, to the extent that women with poor education are 3.5 times more likely to be overweight than the more educated (OECD, 2019). In light of this evidence, there has been a push to study the link between obesity and its most important determinants. A negative gradient between socioeconomic status (SES) and BMI or obesity has been witnessed extensively (e.g., Chou et al., 2004; Lakdawalla & Philipson, 2009; Baum & Ruhm, 2009; Jolliffe, 2011; Esposito et al., 2020), based on several measures of SES, including social class, educational attainment, income, consumption, and ownership of certain household assets, as reflected in a ‘wealth’ index (Wagstaff & Watanabe, 2003).

In this study, we focus on the way income and excess BMI are associated in order to describe the health status of worse-off in comparison to better-off individuals. Income correlates to excess body weight and obesity through various pathways. For instance, wealthy individuals can afford healthier food and more physical activity during leisure time, whereas high income is linked to higher health literacy, which is positively related to health-promoting behaviors (healthy eating, systematic physical activity, etc.). We start by measuring obesity and the degree of income-related inequality in obesity status to observe who is disproportionally affected by it. We then apply standard decomposition methods to reveal the causes of the existing inequalities in obesity that stem from disparities in its determinants. However, to properly evaluate the impact of obesity and account for the long right tail of the BMI distribution (particularly moderate and severe obese statuses), we further compute and decompose inequality indexes in both obesity depth and severity. Finally, we document potential changes in obesity status inequalities over time by comparing two different points in time (i.e., 20-year horizon). Importantly, due to documented substantial health status differences between women and men (Ferretti & Mariani, 2017; Gavurova et al., 2021; Zhang & Wang, 2004), the analysis is performed differentiating by gender.

Many studies have considered possible gender differences in the association between socioeconomic indices and obesity prevalence, as diversity in both biological and social attributes tends to give rise to different health outcomes between genders. One of the first studies evidencing a differential gender pattern is Sobal and Stunkard (1989), who found that obesity is associated with lower SES in women in high-income countries, with no such pattern being observed in men. A large volume of research further identifies the existence of a differential socioeconomic gradient in obesity by gender. Zhang and Wang (2004) claimed that the inverse association between SES and obesity status is stronger in women than in men (and generally weaker in minority groups). Ljungvall and Gerdtham (2010) analyzed SES-related inequalities in obesity using longitudinal data for a Swedish cohort and found inequalities in obesity favoring the rich among women solely, with income being the main driving factor, but the inequality appeared to decline over time. They attributed this finding mainly to increased obesity prevalence, as in absolute terms obesity increased uniformly across income groups. On the basis of absolute and relative inequality indexes, Devaux and Sassi (2013) observed large and persistent social inequalities in obesity and overweight by education level and socioeconomic status in OECD countries, which were larger in women than men.

For the case of Spain, several studies report the existence of SES-related inequalities in obesity to the detriment of the poor (Costa-Font & Gil, 2008; Rodriguez-Caro et al., 2016; Merino Ventosa & Urbanos-Garrido, 2016). Costa-Font et al. (2014) examined cross-country trends in income inequalities in unhealthy lifestyles (obesity, smoking and alcohol intake) between Spain and the UK, and showed that inequality in obesity appeared to increase in Spain by 50% among females from 1987 to 2006. Merino Ventosa and Urbanos-Garrido (2016) provided complementary evidence regarding SES inequalities in obesity, using path analysis to disentangle the direct and indirect effects of SES on obesity. They found significant pro-rich inequality in obesity, particularly for women.

However, a limitation of the above studies (Rodriguez-Caro et al., 2016 is an exception) is their focus on the obesity prevalence, neglecting the distribution of BMI beyond this point,^1^ even though it is well-known that the health risks associated with being obese are worse at the top of the BMI distribution (Jolliffe, 2004, 2011). To overcome this issue, Jolliffe (2004, 2011) slightly modified the Foster-Greer-Thorbecke (FGT) index, which was originally introduced by Foster et al. (1984) to measure poverty. Specifically, Jolliffe set up obesity indices, expressing depth and severity as the excess BMI above the obesity threshold and the squared excess, respectively. This method addresses two important weaknesses of the obesity prevalence measure. Firstly, both indexes are more robust to measurement error near the threshold. Secondly, a distinction between the just slightly overweight or obese and the completely or morbidly obese is possible, which is more insightful in terms of public health policy recommendation. This analysis was further extended by Bilger et al. (2017) who combined the FGT measure with the standard concentration index (CI) to gain further insight on the SES gradient of obesity. Using US NHANES data from 1971 to 2012, they evidenced that income inequality in obesity prevalence had almost disappeared during these years; however, when distribution-sensitive measures of obesity were analyzed, inequalities in depth and severity of obesity appeared to continue disproportionally affecting the poor. The authors also decomposed the FGT-concentration indices (FGT-CIs) into the contribution of the basic factors responsible for overall inequality.

In this study, as already mentioned, we analyze the existence of income-related inequalities in obesity status, depth and severity for Spain. Our study is based on the approach developed by Bilger et al. (2017), which we apply for a European country with very high and rising obesity prevalence rates. For the purposes of our analysis, we use cross-sectional individual-level data gathered from the Spanish National Health Survey (ENSE). We begin by estimating the income-related inequality in obesity status and decompose this metric into its basic determinants (Wagstaff et al., 2003). Next, we use the FGT transformation to our variable of interest (BMI) to estimate the CI of the depth and severity of obesity, ranking the observations according to our SES status variable of choice (income). We then decompose such metrics into their basic determinants.

While this paper extends the literature on income-related inequalities in obesity, our main contribution lies in this study being the first application to European data in the calculation of distribution sensitive measures of obesity. Overall, this study provides novel evidence indicating that inequalities in depth and severity of obesity are also much greater for the poor compared to the rich in and among women in Spain. Moreover, income inequality in obesity has decreased substantially over the past two decades in the case of women. The paper is organized as follows. The method section describes the measurement of the obesity measures and income-related inequality, as well as the decomposition techniques. We continue by presenting the data used for the analysis and present the main results. The final section offers a discussion on the main findings.

## Methods

To define the measures of obesity, we start by using the modified FGT index, as in Jolliffe (2011) and Bilger et al. (2017), as follows:

1 $${Y}_{i}(\alpha )=\left\{\begin{array}{l}{({BMI}_{i}-c)}^{\alpha }, if {BMI}_{i}\ge c\\ 0, otherwise\end{array}\right.$$

where *BMI* is the individual’s body mass index calculated from self-reported weight and height data (weight [kg]/ height [m^2^]), *c* is the obesity threshold and *α* is a parameter weighting the deviation above the obesity cut-off point. According to the well-established WHO criteria, we assume a parameter *c* = 30 kg/m^2^. When $\alpha =0$, $Y\left(0\right)$ is an indicator variable equal to 1 for obese individuals (BMI ≥ 30), providing a measurement of obesity status. When $\alpha =1$, $Y\left(1\right)$ measures how far above 30 the individual’s BMI is for obese individuals, providing a measure of depth of obesity; while when $\alpha =2$, $Y(2)$ yields a severity measure of obesity and is measured as the squared excess BMI over 30.

The next basic step of the analysis is the measurement of income-related inequality in the distribution of the above three obesity measures. We make use of the concentration index (CI), which has become the standard tool for quantifying income-related inequalities in a health measurement (Kakwani, 1977; Kakwani et al., 1997; O’Donnell et al., 2016; Wagstaff & van Doorslaer, 2000; Wagstaff et al., 1991) and is frequently used in the obesity literature (Costa-Font & Gil, 2008; Zhang & Wang, 2004). This index relates the concentration of a health variable with the cumulative rank of the income distribution. It can be computed with individual-level data as follows:

2 $$CI\left(\alpha \right)=\frac{2}{N\mu \left(\alpha \right)} \sum_{i=1}^{N}{Y}_{i}\left(\alpha \right){R}_{i}-1$$

where ${Y}_{i}$ ($\alpha )$ is the health variable of interest of the individual $i$, $\mu \left(\alpha \right)$ denotes its mean value and ${R}_{i}$ is the relative income rank of the person *i*.^2^ The $CI\left( \alpha \right)$ ranges between − 1 and + 1 which enables the comparison of inequality between years and groups. As $Y_{i} \left( \alpha \right)$ corresponds to a measure of bad health, negative values of the $CI\left( \alpha \right)$ indicate that this variable is concentrated among the worse-off, or the inequalities favor high-SES individuals (pro-rich). A zero value of the index represents an equal distribution of obesity across income.

Just in the case of obesity status, since it is a binary measure and because of problems associated with bounded variables, Wagstaff (2005) and Erreygers (2009) propose correcting the associated inequality index to overcome these issues.^3^ While both correction methods satisfy the mirror condition (Erreygers & Van Ourti, 2011), we follow the approach of Costa-Font et al., (2014) and apply the Erreygers’s correction procedure for the $CI\left( 0 \right)$ which is calculated as follows^4^:

3 $$CCI\left( 0 \right) = \frac{4\mu \left( 0 \right)}{{Y\left( 0 \right)^{max} - Y\left( 0 \right)^{min} }}*CI\left( 0 \right)$$

where $Y\left( 0 \right)^{max}$ and $Y\left( 0 \right)^{min}$ denote the maximum and minimum values or bounds of the prevalence of obesity $Y\left( 0 \right)$, respectively, and $CCI\left( 0 \right)$ is the corrected concentration index of obesity status.

In practice, these inequality indexes are calculated based on a convenient regression formula in which a fractional rank variable is created (Kakwani et al., 1997; O’Donnell et al., 2008). In contrast to the CCI (0) for obesity status, the CI(1) for depth and the CI(2) for severity are not only affected by the rank of the income distribution of the individuals who exceed the obesity threshold, but also by the excess of BMI above this threshold.

### Decomposition of inequalities

We apply the standard decomposition of the inequality index into their main contribution factors, which correlate with each of our health variables [$$Y\left( \alpha \right)$$] and income (Wagstaff et al., 2003). This requires a previous regression estimation of the health variable of interest on the set of its *K* determinants ($$X_{k} )$$.

#### Obesity status

According to Wagstaff et al. (2003), the concentration index for obesity status C*CI(0)* can be decomposed into their main contributing factors plus a residual. The concentration index for obesity can be written as follows:

4 $$CCI\left( 0 \right) = \Sigma_{k} \left( {\frac{{\beta_{k} \overline{x}_{\kappa } }}{\mu \left( 0 \right)}} \right)C_{k } + \frac{{GC_{\varepsilon } }}{\mu \left( 0 \right)} = \Sigma_{k} \eta_{k} C_{k } + \frac{{GC_{\varepsilon } }}{\mu \left( 0 \right)}$$

where $\beta_{k}$ is the regression coefficient (marginal effects) of factor *K* on *Y(0)*, $\mu \left( 0 \right)$ is the mean of obesity, $\overline{x}_{k}$ is the mean of $x_{k}$, $C_{k }$ is the concentration index for $x_{k}$, and $GC_{\varepsilon }$ is the generalized concentration index for the error term (*ε*). Alternatively, the *CCI(0)* is equal to a weighted sum of the concentration indices of the *K* regressors, where the weight for $x_{k}$ is the elasticity of obesity with respect to $x_{k} (\eta_{k}$).^5^

#### Obesity depth and severity

Due to a large mass of zero observations (non-obese) and a highly right-skewed distribution of positive values for depth, *Y(1)*, and severity, *Y(2)*, a Two-Part Model (TPM) approach is used instead to estimate the contributions of regressors ($x_{k}$) to these health variables (e.g. Bilger et al., 2017; Duan et al., 1983; Jones, 2010; Pohlmeier & Ulrich, 1995). Regarding the specification of the first part of the TPM, we apply a Logit model and, for the second part, we choose a generalized linear model (GLM), in line with Nelder and Wedderburn (1972), as this family offers many alternatives to the linear model that are suitable for skewed data.^6^ Afterwards, we use the Van Doorslaer et al. (2004) approximation of the Wagstaff et al. (2003) decomposition of the concentration indexes for obesity depth and severity as follows:

5 $$CI\left( \alpha \right) = \mathop \sum \limits_{k = 1}^{k} \frac{{\partial \overline{\varepsilon }\left( {Y\left( \alpha \right){|}X} \right)}}{{\partial x_{k} }} \frac{{\overline{x}_{\kappa } }}{\mu \left( \alpha \right)}C_{k } + GC_{\varepsilon } = \mathop \sum \limits_{k = 1}^{k} \eta_{k} C_{k } + GC_{\varepsilon }$$

where $\alpha = 1,2$ so *CI(1)* is the inequality index of obesity depth and *CI(2)* is the inequality index of obesity severity, $C_{k }$ is the CI of the factor *X*_*k*_, $\mu \left( \alpha \right)$ is the sample average of $Y\left( \alpha \right)$, $\overline{x}_{\kappa }$ the sample average of factor $x_{k}$, $\frac{{\partial \overline{\varepsilon }(Y\left( \alpha \right)|X)}}{{\partial x_{k} }}$ the average marginal effect of factor $x_{k}$ on $Y\left( \alpha \right)$ obtained from the TPM estimates and, finally $GC_{\varepsilon }$ is the generalized CI of the regression residuals.^7^

### Oaxaca-blinder decomposition

In addition to utilizing methods for disentangling the main determinants of health inequalities, we further investigate the changes in inequalities in obesity status in Spain over the period 1997–2017, aiming to contribute to the debate on the drop in income-related inequality in obesity over time for women. We follow the approach of Wagstaff et al. (2003) and apply an Oaxaca-Blinder decomposition to Eq. (4). If we denote the elasticity of Y(0) with respect to *x*_*k*_ at time *t* by *η*_*kt*_ and apply the Oaxaca’s method, we get:

6 $$\Delta CCI\left( 0 \right) = \sum \eta_{kt} *\left( {C_{kt} - C_{kt - 1} } \right) + \sum C_{kt - 1} *\left( {\eta_{kt} - \eta_{kt - 1} } \right) + {\Delta }\left( {GC_{\varepsilon t} /\mu_{t} } \right)$$

with the alternative being:

7 $$\Delta CCI\left( 0 \right) = \sum \eta_{kt - 1} *\left( {C_{kt} - C_{kt - 1} } \right) + \sum C_{kt} *\left( {\eta_{kt} - \eta_{kt - 1} } \right) + {\Delta }\left( {GC_{\varepsilon t} /\mu_{t} } \right)$$

This approach allows us to see the extent to which the changes in health inequalities over time are due to changes in inequality in the determinants of health, rather than to changes in their elasticities. However, the drawback of this procedure is that it does not allow us to investigate inter-temporal changes in *CCI(0)* occurring within the elasticity, $\eta_{k}$, mainly when their components may vary in different directions (Wagstaff et al., 2003).

## Data

We base our analysis on cross-sectional data from the Spanish National Health Survey (ENSE) for 1997 and 2017. The ENSE is a periodic study conducted by the Ministry of Health, Consumption and Social Welfare and collects health information on the entire population on health status, personal, social, and environmental determinants of health, and the use and access to health services. The weight and height of the respondents is self-reported and this information is used to calculate the individual BMI.^8^ We use household income to compute equivalent net income. In order to deal with the potential selectivity bias due to the non-randomness of non-reporting in the household income variable when missing, we replace missing income values with imputed ones based on a linear interpolation of income on a set of SES status and demographic characteristics of the individual. The income figure is then transformed into its natural logarithm, which is used as our rank variable.^9^

A total of 29,195 individuals, corresponding to 23,089 adults (15 and over) and 6,106 minors (0–14 years old), were interviewed in the 2017 survey. In this study, we restrict the sample and solely consider individuals aged 18 to 64 years old with complete information on the relevant variables. Old-age individuals are disregarded to reduce the bias arising through greater mortality among the more obese as well as the measurement error affecting self-declared weight and height (and hence BMI) that tends to rise with age (Gil & Mora, 2011). We further discard those with no information on either weight or height and are left with a sample N = 15,093 observations for 2017. As for 1997, under the same assumptions, we ended up with a sample of N = 4508 individuals.

We decompose the previously estimated concentration indices of obesity status *CCI(0)*, depth *CI(1)* and severity *CI(2)* into their determining factors differentiating by gender. We use dummies of age cohorts, marital status, employment status, completed level of education (i.e., primary or no education, secondary education and university education), equivalent net monthly income (log), daily smoking and a set of dummies for region of residence as basic predictors (see Table 6 in the Appendix).

## Results

**Fig. 1:**
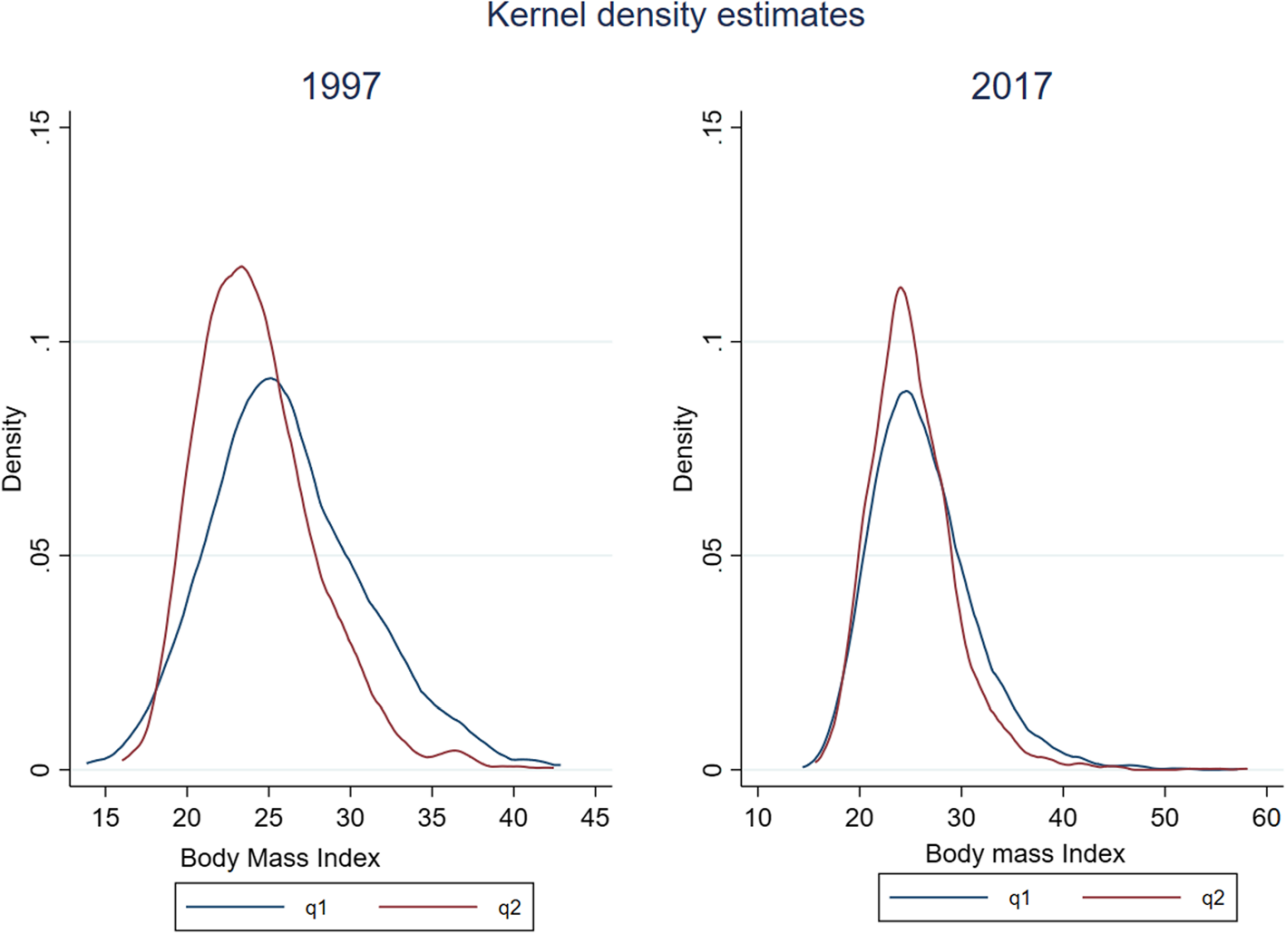
Distribution of BMI over time and by income quintile. Notes: q1 denotes the lowest income quintile, q2 denotes the highest income quintile

Figure 1 shows the BMI distribution of individuals belonging to the lowest income quartile versus the BMI distribution of those belonging to the highest income quartile for both years (1997 and 2017). A thorough understanding of the relationship between income and body weight can provide useful insights for developing effective intervention programs and policies. The figures clearly indicate that there are important differences in the shape of the distributions between the two populations mainly in 1997, with the BMI distribution of the worse-off being less peaked, indicating greater spread in the tails and as expected, shifted towards the right. Comparing the two points in time, we notice that the shape of the two distributions appears to become more similar intertemporally, resulting in just slight differences between the two populations in 2017 (distribution of the worse-off has a slightly higher dispersion than the one of the better-off individuals) in contrast to 1997, where differences in shapes are much more pronounced.

### Income-related inequality of obesity status, depth and severity

Table 1 reports the calculation of the three income-related inequality indexes of obesity. Several key results emerge from these findings. Firstly, our estimates confirm that obesity status is concentrated among the poor for both genders and years in Spain, as the *CCI(0)* is negative and statistically significant in all cases. Secondly, the degree of inequality in obesity status has a strong declining trend among women only, although the prevalence of obesity in the Spanish population rises continuously throughout these years. These trends are in line with previous findings in the literature and specifically complement those reported by Costa-Font et al. (2014). Interestingly, compared to the evidence shown by Costa-Font et al. (2014) our estimates suggest the existence of rising inequalities among women peaking around 1997 [*CCI(0)* =  − 0.125] and then declining until the mid-2000s (Costa-Font et al., 2014) and remaining fairly constant thereafter at a *CCI(0)* = − 0.085 level in 2017. Thirdly, we contribute by reporting the existence of significant and large income-related inequalities in obesity depth, *CI(1)*, and severity, *CI(2)*, to the detriment of the poor and for both genders. When looking just at individuals above the obesity threshold, we find evidence that depth and severity measures of obesity are concentrated among low-income groups. Our data also indicate a gender pattern, that is, larger inequalities in depth and severity among women and for both years, but also an intertemporal declining trend basically among the female population.

**Table 1:** Income-related inequalities in Obesity Status, Depth and Severity by gender, 1997 and 2017

Women			Men		
Status CCI(0)	Depth CI(1)	Severity CI(2)	Status CCI(0)	Depth CI(1)	Severity CI(2)
1997					
− 0.125*** (0.035)	− 0.271*** (0.046)	− 0.284*** (0.083)	− 0.056*** (0.015)	− 0.144** (0.060)	− 0.132*** (0.015)
2017					
− 0.085*** (.018)	− 0.173*** (0.016)	− 0.191*** (0.045)	− 0.056*** (0.014)	− 0.112*** (0.026)	− 0.116*** (0.065)

*Source* ENSE (1997,2017), Instituto Nacional de Estadística (INE). Inequality in obesity status is measured by the Erreygers Concentration Index CCI(0). Standard errors in parentheses. *p < 0.10; **p < 0.05; ***p < 0.01

### Decomposition of income-related inequalities

**Fig. 2:**
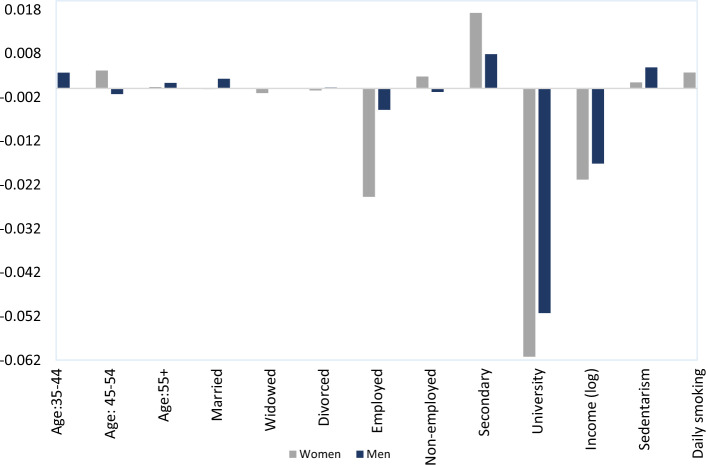
Decomposition of the Erreygers Inequality index of Obesity Status by gender, 2017

Figure 2 presents the decomposition of income-related inequality in obesity status, *CCI(0)*, for women and men in 2017 (see Appendix Figs. 3 and 4 for similar figures including the comparison with the year 1997). Note that the vertical axis represents the contribution of each determinant *k* to the overall inequality. Obesity is associated with lower educational attainment, employment status and income (negative elasticity), with the corresponding estimated coefficients $\beta_{k}$ being sizable and negative. As expected, those regressors contribute towards increasing the CCI(0) in women (as shown in Table 7), as they are concentrated among the better-off individuals (C_Ik_ > 0). According to Fig. 2, the largest part of the income-related inequality in obesity status for women is driven by SES factors and, specifically, by university education (− 0.061 which translates to 43% of the total contribution), employment (− 0.025 which translates to 17% of the total contribution) and income (− 0.021 which contributes to 14% of the total contribution). Similarly, inequalities in obesity status among men, as measured by the CCI(0), are almost exclusively explained by university education (− 0.051 which translates to 43%) and income (− 0.017 which is the 14% of the total contribution). Interestingly, unhealthy habits like smoking and a sedentary lifestyle contribute towards lowering inequalities in obesity status in both genders, although in an opposed way. While sedentary behavior (daily smoking) is concentrated among the better-off (worse off), it is significant and positively (negatively) correlated with obesity status. Nevertheless, the contribution of lifestyles to overall inequality is modest (around 6%). Detailed estimates can be found in Table 7 (Appendix).

**Fig. 3:**
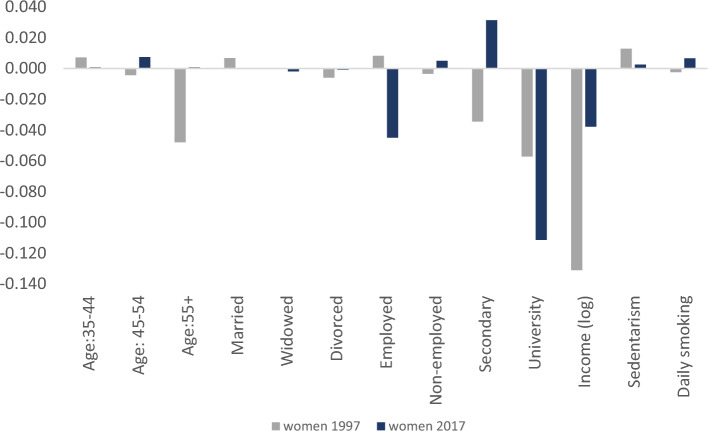
Decomposition of the Erreygers Inequality index of Obesity Status, Women

**Fig. 4:**
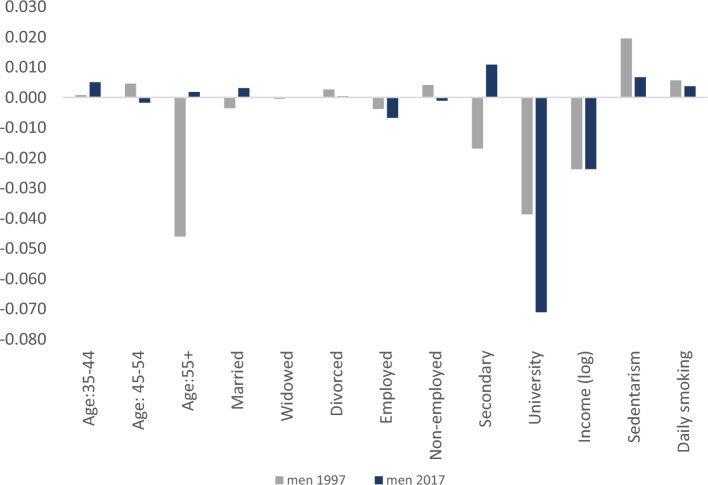
Decomposition of the Erreygers Inequality index of Obesity Status, Men

Tables 2 and 3 show the estimates of the decomposition of the FGT-CIs for obesity depth, *CI(1)*, and severity, *CI(2)*, according to Eq. (5) for women and men, respectively. Table 2 reveals that mainly university education (46%), then employment (19%) and, to a lesser extent, income (7.5%) are again the main contributors to the emergence of inequalities in excess of obesity. With respect to inequalities of severity, university education also turns out to be the most significant individual contributor (around 39%), followed by employment (17%) and income (around 11%). Table 3 shows that the main determinants of rising inequalities in excess of obesity for men are university education (around 46%) and income (around 7%). A similar pattern is observed for inequalities in severity of obesity, with men of low SES being worst affected: the contribution of university education is around 38% and, of income, around 11.6%. While employment status has a minor role in explaining inequalities among men, sedentary behavior tends to reduce inequalities for both depth (around 8%) and severity (around 10%).

**Table 2:** Decomposition: Inequality of Depth and Severity of Obesity, Women, 2017

	*CI*_*K*_	Depth—CI(1)			Severity—CI(2)		
		*η*_*k*_	*CIY*_*k*_	%	*η*_*k*_	*CIY*_*k*_	%
Age 35–44	0.023	0.024	0.001	0.2	0.004	0.000	0.0
Age 45–54	0.074	0.082	0.006	1.7	0.093	0.007	1.5
Age 55+	− 0.014	0.011	0.000	0.0	− 0.011	0.000	0.0
Married	0.080	0.038	0.003	0.9	0.042	0.003	0.8
Widowed	− 0.158	0.020	− 0.003	0.9	0.014	− 0.002	0.5
Divorced	− 0.189	0.028	− 0.005	1.5	0.013	− 0.003	0.6
Employed	0.239	− 0.277	− 0.066	18.8	− 0.318	− 0.076	17.2
Non-employed	− 0.167	− 0.069	0.012	3.3	− 0.157	0.026	5.9
Secondary	− 0.069	− 0.316	0.022	6.2	− 0.336	0.023	5.2
University	0.462	− 0.349	− 0.161	45.9	− 0.378	− 0.175	39.5
Income (log)	0.043	− 0.616	− 0.026	7.5	− 1.161	− 0.050	11.2
Sedentarism	0.100	0.182	0.018	5.2	0.285	0.029	6.5
Daily smoking	− 0.051	− 0.120	0.006	1.8	− 0.127	0.007	1.5
Residuals			0.026			0.022	
Sum			− 0.200			− 0.213	
CI			− 0.173			− 0.191	

*Source* ENSE (2017), Instituto Nacional de Estadística (INE). CIk: concentration index of factor k, η_k_: elasticity of the FGT measure Y with respect to factor k, CIYk: contribution made by factor k to the overall FGT-CI, CI: concentration index. 0.062% of the total contribution is attributed to the region of residence (Autonomous community) which is not presented in the table for space saving purposes

**Table 3:** Decomposition: Inequality of Depth and Severity of Obesity, Men, 2017

	*CI*_*K*_	Depth—CI(1)			Severity—CI(2)		
		*η*_*k*_	*CIY*_*k*_	%	*η*_*k*_	*CIY*_*k*_	%
Age 35–44	0.070	0.085	0.006	2.7	0.125	0.009	3.4
Age 45–54	− 0.004	0.130	− 0.001	0.2	0.167	− 0.001	0.3
Age 55+	0.005	0.061	0.000	0.1	0.056	0.000	0.1
Married	0.022	− 0.053	− 0.001	0.5	− 0.301	− 0.007	2.6
Widowed	− 0.056	− 0.002	0.000	0.0	− 0.013	0.001	0.3
Divorced	− 0.008	− 0.013	0.000	0.0	− 0.049	0.000	0.1
Employed	0.178	− 0.070	− 0.012	5.7	− 0.037	− 0.007	2.6
Non-employed	− 0.111	0.083	− 0.009	4.2	0.112	− 0.012	4.8
Secondary	− 0.040	− 0.195	0.008	3.6	− 0.193	0.008	3.0
University	0.554	− 0.181	− 0.100	46.1	− 0.179	− 0.099	38.5
Income (log)	0.040	− 0.409	− 0.017	7.6	− 0.738	− 0.030	11.5
Sedentarism	0.100	0.174	0.017	8.0	0.251	0.025	9.7
Daily smoking	− 0.093	− 0.047	0.004	2.0	− 0.056	0.005	2.0
Residuals			0.008			0.006	
Sum			− 0.120			− 0.122	
CI			− 0.112			− 0.116	

*Source* ENSE (2017), Instituto Nacional de Estadística (INE). CIk: concentration index of factor k, ηk: elasticity of the FGT measure Y with respect to factor k, CIYk: contribution made by factor k to the overall FGT-CI, CI: concentration index. 0,190% of the total contribution is due to the region (Autonomous community) which is not presented in the table for space saving purposes

Overall, the evidence shown by these tables appear to demonstrate that education is the single most relevant determinant in explaining inequalities for all measures of obesity and both genders, with the contribution becoming less preponderant as one moves from status to depth and severity.

### Comparing drivers of inequalities overtime

Given the reported declining trend in income-related inequalities in obesity status among women when comparing the data in 1997 and 2017 (Table 1), in Table 4, we investigate the main responsible drivers behind these inequalities, in line with Wagstaff et al. (2003). The column of the table named “change” shows the change of each determinant in explaining the variation in inequalities across these years. Notice that a positive (negative) value means a contribution towards a reduction (increase) in the obesity status inequality. Our findings indicate that the decrease in the inequality in obesity status among women between 1997 and 2017 is basically due to changes with respect to income (0.039) and secondary education (0.033). In the opposite direction, indicating a pro-rich inequality change, were the changes that occurred in relation to university education (− 0.035) and employment status (− 0.028).

**Table 4:** Inequality decompositions of obesity status for 1997 and 2017, and change 1997–2017, Women

	η_k_		CI_k_		CIY_k_		Change	%
	1997	2017	1997	2017	1997	2017		
Age 35–44	0.080	0.080	0.041	0.004	0.003	0.000	− 0.003	− 0.29
Age 45–54	0.154	0.117	− 0.013	0.035	− 0.002	0.004	0.006	0.61
Age 55+	0.155	0.060	− 0.140	0.005	− 0.022	0.000	0.022	2.21
Married	0.282	− 0.003	0.011	0.049	0.003	0.000	− 0.003	− 0.32
Widowed	0.000	0.008	− 0.245	− 0.139	0.000	− 0.001	− 0.001	− 0.11
Divorced	0.018	0.004	− 0.156	− 0.122	− 0.003	0.000	0.002	0.23
Employed	0.203	− 0.205	0.018	0.121	0.004	− 0.025	− 0.028	− 2.84
Non-employed	0.069	− 0.018	− 0.023	− 0.153	− 0.002	0.003	0.004	0.43
Secondary	− 0.177	− 0.313	0.088	− 0.055	− 0.016	0.017	0.033	3.29
University	− 0.107	− 0.257	0.242	0.238	− 0.026	− 0.061	− 0.035	− 3.53
Income (log)	− 3.114	− 0.838	0.019	0.025	− 0.059	− 0.021	0.039	3.86
Sedentarism	0.062	0.022	0.093	0.062	0.006	0.001	− 0.004	− 0.44
Daily smoking	− 0.025	− 0.095	0.044	− 0.038	− 0.001	0.004	0.005	0.47
Total	–	–	–	–	− 0.119	− 0.081	0.038	–

*Source* ENSE (1997, 2017), Instituto Nacional de Estadística (INE). η_k_: elasticity of y with respect to factor k, CIk: concentration index of factor k, CIYk: contribution made by factor k to the overall inequality. Dummies for each autonomous community are included but are not shown for space saving purposes

As we are further interested in identifying the specific drivers behind this pattern, we applied the Oaxaca-Blinder decomposition analysis shown in Eqs. (6) and (7), designed to identify whether the changes in inequality in obesity status over time are due to changes in the elasticity or changes in the inequality of each determinant. Interestingly, Table 5 (last row) shows that, overall, the variation in inequalities in the determinants of obesity are, by far, much greater than the changes in the elasticities of obesity. More specifically, while income and secondary education appear to be the two fundamental factors explaining the decrease in *CCI(0)* index, in the case of the income variable, this effect is explained by a huge decrease in the elasticity of obesity [i.e., a change of 0.043 according to Eq. (6) or a change of 0.056 according to Eq. (7)] while the CI of income remains fairly constant, whereas the effect of secondary education is explained by a large decrease of the CI of this determinant [i.e., 0.045 according to Eq. (6) or 0.025 according to Eq. (7)], so that secondary education among women turns out to be pro-poor in 2017. In contrast, our estimates reveal that university education and employment contribute towards raising the *CCI(0)* score, although in a distinct way again. We document a huge increase in the negative impact of the elasticity of university education on obesity during this period [i.e., a change in this elasticity of − 0.036 in both equations] while the CI of university education hardly varied, whereas the positive contribution of employment on *CCI(0)* is due to a significant rise in the pro-rich inequality distribution of employment among women over the course of these years [i.e., a change of − 0.021 or − 0.049 according to Eqs. (6) or (7), respectively].

**Table 5:** Oaxaca-type decomposition for change in inequality, Women, 1997–2017

	Equation (6)		Equation (7)		Total
	Change inequality regressors (ΔC*η)	Change elasticity of obesity (Δη*C)	Change inequality regressors (ΔC*η)	Change elasticity of obesity (Δη*C)	
Age 35–44	− 0.003	0.000	− 0.003	0.000	− 0.003
Age 45–54	0.006	0.000	0.007	− 0.001	0.006
Age 55+	0.009	0.013	0.023	− 0.001	0.022
Married	0.000	− 0.003	0.011	− 0.014	− 0.003
Widowed	0.001	− 0.002	0.000	− 0.001	− 0.001
Divorced	0.000	0.002	0.001	0.002	0.002
Employed	− 0.021	− 0.007	− 0.049	0.021	− 0.028
Non-employed	0.002	0.002	− 0.009	0.013	0.004
Secondary	0.045	− 0.012	0.025	0.007	0.033
University	0.001	− 0.036	0.000	− 0.036	− 0.035
Income (log)	− 0.005	0.043	− 0.018	0.056	0.039
Sedentarism	− 0.001	− 0.004	− 0.002	− 0.003	− 0.004
Daily smoking	0.008	− 0.003	0.002	0.003	0.005
Total	0.040	− 0.002	0.064	− 0.026	0.038

*Source* ENSE (1997,2017), Instituto Nacional de Estadística (INE). Dummies for each autonomous community are included but are not shown for space saving purposes

## Conclusions

The aim of this paper was threefold. Firstly, we measured income-related inequality in obesity and decomposed it into its main determinants, aiming to bring to light important information regarding the way income and BMI are related to one another in Spain, one of the European countries with the highest levels and rapid increase of obesity prevalence. Secondly, in an attempt to move a step further and examine this same relationship beyond the obesity threshold, we measured and further decomposed inequalities in the depth and severity of obesity into their main factors. To the best of our knowledge, no other previous studies have measured the latter inequalities for a European country, making this one of the main contributions of this work. Thirdly, we investigated the potential changes in obesity status inequality over time among women and further performed an Oaxaca-Blinder decomposition analysis to attribute those changes into their main factors.

Our results indicate that income-related inequalities in the three measures of obesity status, depth and severity are to the disadvantage of the poor and much larger among women. Moreover, even though all three measures of obesity have been increasing among the Spanish population over time (OECD, 2019), we documented decreasing trends in all these inequality indexes, fundamentally among women. However, despite these declining trends, we found that inequalities in both depth and severity remained sizable. Overall, the evidence documented in this study is in line with previous findings for the US economy (Bilger et al., 2017), further indicating that the universal nature of the Spanish healthcare system per se is not a fundamental driver of these inequalities.

Interestingly, the decomposition analysis appears to show that university education is the single most relevant determinant explaining inequalities in the three measures of obesity and for both genders. This is explained by the fact that higher education is much more prevalent among the highest income deciles and education attainment is negatively associated with the three measures of obesity. In other words, if higher education -and to a lower extent employment and income- were more equally distributed across the income distribution, then income-related inequalities in obesity status, depth and severity would be greatly reduced. This result is clearly consistent with previous evidence highlighting the protective role of education on health outcomes and risky health behaviors (Brunello et al., 2016; Clark & Royer, 2013; Kenkel, 1991). The important role of education in shaping obesity in the Spanish female population is also highlighted by Di Paolo et al. (2020), with the authors suggesting that regional policymakers should design policies aimed at reducing school dropout and improving education quality, also through the introduction/improvement of health education programs during the first stages of the education process. Furthermore, our finding of larger income-related inequalities in obesity status for women across the period analyzed, also confirms previous evidence in the literature (Zhang & Wang, 2004; Ljungvall & Gerdtham, 2010; Costa-Font et al., 2014). However, this study contributes by showing larger inequality indexes in both obesity depth and severity, also to the detriment of less advantaged women.

The finding of a differential gender impact, that is, larger inequalities in the three measures of obesity status, depth and severity among women, seems to result basically from the role of employment, as this determinant is found to be more pro-rich distributed among women in Spain. Other researchers also mention the greater tendency of women to be more prone to societal changes towards a thinner body image than men (Bilger et al., 2017). Those larger disparities in depth and severity of obesity are in line with findings arguing that highest levels of BMI are often observed among the worse-off and poorly educated and more generally among those in disadvantaged socioeconomic circumstances.

When analyzing the factors responsible for the declining trend in income-related inequalities in obesity status among women over the course of the period 1997–2017, we found that income and secondary education played a major role. With respect to income, this is explained by a large decrease in the income elasticity of obesity, whereas, for secondary education, there was a substantial decrease in the inequality level, so secondary education among women turned out to be pro-poor.

Overall, income-related inequalities in obesity are still a reality in Spain especially in terms of the depth and severity of obesity and mainly among women, something that could aggravate the socioeconomic gradient in health even further. The findings suggest that a more equal distribution of higher education, employment, and income would likely reduce those income related inequalities. Reinforcing investment in public education may contribute to the dual objective of reducing inequalities in obesity and achieving a more equitable distribution of income. Thus, focusing attention towards increasing public education budgets to improve human capital and tackle the obesity epidemic while focusing on promoting equal access to education may be worthwhile considering. For instance, policies aimed to improve access to higher education for students from disadvantaged backgrounds (e.g. scholarships, grants) could help reduce those disparities between different income groups. Further, focusing on early childhood education in terms of improving its quality and accessibility, particularly for children from low-income families, is also expected to have long-term positive effects on educational attainment, employment opportunities, and health outcomes. In a similar vein, fostering inclusive educational environments that value and respect diversity, with policies and initiatives that promote inclusivity, would probably lead to better outcomes as well, given that they would ensure equal opportunities for students from all backgrounds. Comprehensive career counseling and guidance services to students, particularly those from disadvantaged backgrounds, could address systemic barriers and help students make informed decisions about their educational and career paths, increasing their chances of success, and resulting in lower income-related disparities. Importantly, monitoring disparities in educational outcomes and how these translate to differential health outcomes by regularly collecting and analyzing data is crucial, in the effort to address them, as well as to evaluate whether already implemented interventions that are targeted in reducing income-related inequalities are effective or not.

## Data Availability

All the data used in the empirical analysis are publically available and can be downloaded from the following website: https://www.sanidad.gob.es/estadEstudios/estadisticas/encuestaNacional/..

## Appendix

See Tables 6, 7 and Figs. 3, 4.

**Table 6:** Description of variables

	Description
*Dependent variables*	
Obesity	1 when BMI > = 30, 0 otherwise
Depth	excess BMI beyond the obesity threshold
Severity	squared excess BMI beyond the obesity threshold
*Independent variables*	
**Demographics**	
Age 18–34	1 when aged 18–34, 0 otherwise
Age 35–44	1 when aged 35–44, 0 otherwise
45–54	1 when aged 45–54, 0 otherwise
Age 55 +	1 when aged 55+, 0 otherwise
Married	1 when married, 0 otherwise
Widowed	1 when widowed, 0 otherwise
Divorced	1 when divorced/separated, 0 otherwise
**SES**	
Primary	1 when primary education the highest level achieved, 0 otherwise
Secondary	1 when secondary education the highest level achieved, 0 otherwise
University	1 when tertiary education the highest level achieved, 0 otherwise
Employed	1 when employed, 0 otherwise
Non-employed	1 when unemployed, 0 otherwise
Inactive	1 when inactive, 0 otherwise
Income(log)	equivalent net monthly income in logs
**Lifestyle**	
Sedentarism	1 if working in a sedentary job, 0 otherwise
Daily smoking	1 if daily smoker, 0 otherwise

**Table 7:** Decomposition of the Erreygers Inequality Index of Obesity Status, 2017

	Women, 2017				Men, 2017			
	*η*_*k*_	*CI*_*K*_	*CIY*_*k*_	%	*η*_*k*_	*CI*_*K*_	*CIY*_*k*_	%
Age 35–44	0.080	0.004	0.000	0.2	0.098	0.037	0.004	3.0
Age 45–54	0.117	0.035	0.004	2.8	0.141	− 0.009	− 0.001	1.1
Age 55 +	0.060	0.005	0.000	0.2	0.103	0.012	0.001	1.0
Married	− 0.003	0.049	0.000	0.1	0.077	0.028	0.002	1,8
Widowed	0.008	− 0.139	− 0.001	0.7	0.000	− 0.065	0.000	0.0
Divorced	0.004	− 0.122	0.000	0.3	− 0.007	− 0.032	0.000	0.2
Employed	− 0.205	0.121	− 0.025	17.2	− 0.043	0.114	− 0.005	4.0
Non-employed	− 0.018	− 0.153	0.003	1.9	0.008	− 0.101	− 0.001	0.7
Secondary	− 0.313	− 0.055	0.017	12.0	− 0.151	− 0.052	0.008	6.5
University	− 0.257	0.238	− 0.061	42.6	− 0.130	0.394	− 0.051	42.5
Income (log)	− 0.838	0.025	− 0.021	14.5	− 0.559	0.031	− 0.017	14.2
Sedentarism	0.022	0.062	0.001	1.0	0.065	0.074	0.005	4.0
Daily smoking	− 0.095	− 0.038	0.004	2.5	− 0.043	− 0.062	0.003	2.2
Residuals		− 0.004					0.007	
Sum		− 0.081					− 0.063	
CCI(0)		− 0.085					− 0.056	

*Source* ENSE (2017), Instituto Nacional de Estadística (INE). η_k_: elasticity of y with respect to factor k, CI_k_: concentration index of factor k, CIY_k_: contribution made by factor k to the overall inequality. CCI(0): Corrected Concentration Index. The Dummies for each autonomous community are included but are not shown for space saving purposes

## Footnotes

1 A continuous BMI measure which is converted into a discrete outcome using a standard cut-off point (i.e., BMI > 30) is translated to a loss of important information (Jolliffe, 2004).

2 Notice that the CI is a measure of relative inequality, so that a small change in everyone’s obesity (health) leaves the CI index unchanged.

3 These drawbacks have previously been discussed in the literature and include: (1) problems associated to the minimum and maximum values of the CI calculated on the basis of a binary variable that depend upon the mean of this variable making comparison of populations with different mean health levels problematic (Erreygers, 2009); (2) the non-satisfactory nature of the mirror property (Erreygers et al., 2012); (3) the arbitrary nature of the index in cases where the health variable has a qualitative nature; and (4) when the health variable is binary, the limits of the CI are not necessarily − 1 and + 1 (Wagstaff, 2005).

4 The Erreygers corrected CI has been found insensitive to any feasible equal addition to the health variable (Erreygers and Van Ourti, 2011).

5 The residual component should approach zero for a well-specified model.

6 We used the Box-Cox (1964) and Park (1966) tests to determine the GLM link function and distribution family.

7 These calculations are based on the Stata code provided by Bilger et al. (2017).

8 Correcting formulas for Spain provided by Gil and Mora (2011) are used to make sure the data do not suffer from significant measurement error.

9 Household earnings are measured as a categorical variable with 8 response categories in our datasets. We employ an interval regression model based on information of the head of the household (age, gender, education, SES and region of residence) to obtain a continuous income measure. Once predicted, we divide it by an equivalence factor (equal to the number of household members elevated to 0.5) to adjust for differences in household size.

## Code availability (software application or custom code)

All the codes used for the empirical analysis can be made available upon request to the authors in case of acceptance.
